# A 12-Year Comparison of Alzheimer’s Dementia Patients With Their Informants in Taiwan

**DOI:** 10.1177/15333175231218089

**Published:** 2023-11-28

**Authors:** Kai-Ming Jhang, Wen-Fu Wang, Kuang-Nan Hsu, Shang-Chien Huang, Sheng-Hsiang Yang, Ling-Chun Huang, Yuan-Han Yang

**Affiliations:** 1 Department of Neurology, 36596 Changhua Christian Hospital, Changhua, Taiwan; 2 Department of Neurology, Taitung MacKay Memorial Hospital, Taitung, Taiwan; 3 Department of Psychiatry, 59084 Tungs' Taichung MetroHarbor Hospital, Taichung, Taiwan; 4 Department of Neurology, 38018 Chi-Mei Medical Center, Tainan, Taiwan; 5 Department of Neurology, 89234 Kaohsiung Medical University Hospital, Kaohsiung Medical University, Kaohsiung, Taiwan; 6 Neuroscience Research Center, Kaohsiung Medical University, Kaohsiung, Taiwan; 7 Department of Neurology, Kaohsiung Municipal Ta-Tung Hospital, 89234 Kaohsiung Medical University Hospital, Kaohsiung, Taiwan; 8 School of Post-Baccalaureate Medicine, College of Medicine, Kaohsiung Medical University, Kaohsiung, Taiwan

**Keywords:** Alzheimer's disease, uniform data set, registration, Taiwan, informant, living situation, family history

## Abstract

**Background:**

To update the characteristics of patients with Alzheimer’s disease (AD) and their informants in Taiwan and compare them from 12 years ago.

**Methods:**

1218 patients with AD and their informants were recruited from six hospitals in Taiwan. The uniform data set version 3.0 (UDS3, form A1-A3) were administered.

**Results:**

Compared with the first registration from 2010-2012 (n = 691), the mean clinical dementia rating sum of boxes score was significantly lower, more patients living independently, and more informants not living together with the patients. A total of 11.2%, 4.1%, 12.8%, and 0.5% of the patients had a reported history of cognitive impairment in their mothers, fathers, siblings, and children, respectively.

**Conclusion:**

Compared with the data from 2010, patients have been diagnosed at a milder disease stage, and their informants used telephone contact more frequently instead of living with the patients. Family histories of cognitive impairment in patients with AD remain frequent.

## Introduction

The number of individuals with dementia is increased worldwide. According to the World Alzheimer Report 2015, 46.8 million people were living with dementia in 2015 and this number is expected to double every 20 years.^1^ Taiwan has become an “aged society” since 2018 and is predicted to become a super-aged society by 2025. The number of patients with dementia has increased dramatically with the rapid increase in the elderly population. The age-stratified prevalence rate approximately doubles every 5 years after the age of 50 years, and the prevalence of dementia was estimated to be 7.54% in the population aged >65 years in Taiwan.^2,3^

Alzheimer’s disease (AD) accounts for approximately 60% of all dementia in Taiwan.^4^ Using the Uniform Data Set (UDS) of the National Alzheimer's Coordinating Center database,^5^ the demographic characteristics of patients with AD and their informants were reported from 2010 to 2012 in Taiwan.^6^ Family histories were also recorded, with 6.7%, 2.7%, and 13.1% of mothers, fathers, and siblings, respectively, having a reported history of dementia. Collaborative research across multiple sites in different countries is accessible in a uniform data format. The clinical picture for patients and informants, as well as family history, is very different among Asian countries.^7,8^

In 2015, the US Alzheimer’s Disease Centers (ADC) implemented Version 3 of the Uniform Dataset (UDS3).^9^ The recoding of demographic data in UDS3 was comparable to that in the first version. However, a more detailed family history was recorded, such as the etiological diagnosis and evaluation method of family members reported to have cognitive impairment. Renewing the most recent characteristics of AD and their informants provides clues for understanding the status of the clinical picture of AD in Taiwan. We then administered the second registration from 2022 to 2023 to obtain updated data on patients with AD and their informants and compared them with the first evaluation.

## Methods

### Measurement

The present registration selected A1 to A3 components from UDS version 3.0 and enrolled six hospitals in Taiwan (one from the north, two from the middle, two from the south, and one from the east of Taiwan). The A1 component included subject demographics, including sex, education, living situation, and marital status. The A2 component was informant demographics, including the relationships and visits between the informant and the subject. There were no major changes in the A1 and A2 components compared with UDS version 1.0. The A3 component represents a family history of cognitive dysfunction. The cognitive status of the participants’ parents, children, and siblings, including mild cognitive impairment (MCI) (single or multiple, amnesic or non-amnesic domain) or dementia (specific etiology coded if known), was recorded. UDS 3.0 added family history of AD or frontotemporal dementia (FTD) mutations and recorded associated neuropsychiatric diagnoses (such as amyotrophic lateral sclerosis (ALS), parkinsonism, or stroke) in the A3 component.^9^

### Participants

All subjects were recruited between March 1, 2022 and January 30, 2023, from six sites: a regional hospital in the north, one regional hospital and one medical center in the middle, one regional hospital and one medical center in the south, and one regional hospital in the east of Taiwan. All subjects underwent a comprehensive medical evaluation, including clinical history, physical and neurological examinations, neuropsychological tests including the Cognitive Ability Screening Instrument (CASI)^10^ and Clinical Dementia Rating (CDR) scale,^11^ brain imaging, and blood chemistry examinations, to exclude other possible causes of the current cognitive status. The Mini-Mental State Examination (MMSE) score used in the present study was converted from the CASI, which has been reported to have a moderate to excellent equivalence with the original MMSE score.^12^ Diagnosis of AD was based on the NINCDS-ADRDA criteria.^13^ Patients with other conditions that may have contributed to the diagnosis of dementia were excluded.

All procedures were approved by the institutional review boards (IRB) of the six sites, and written informed consent was obtained from all participants or their legal representatives. For each recruited subject, a series of neuropsychological assessments, including the CASI and CDR, were evaluated by neuropsychologists, and the clinical history and information of the subjects and informants were recorded by trained study assistants.

### Statistical Analysis

All data collected in the present study were compared to the first AD registration in Taiwan.^6^ All statistical analyses were performed using R Statistical Software (version 4.1.0; R Foundation for Statistical Computing, Vienna, Austria).^14^ All statistical tests were two-tailed and an alpha value of .05 was taken to indicate significance. Descriptive statistical analyses were conducted for all continuous variables with mean ± standard deviation. Differences between categorical data were calculated using Pearson’s chi-square test or Fisher’s exact test. Numerical data were analyzed using Student’s t-test or the Kruskal–Wallis rank-sum test.

## Results

A total of 1218 patients with AD were enrolled and compared with 691 patients with AD in 2010-2012. Table 1 shows the basic characteristics of the patients and informants: 65.4% patients were female, with mean age of the patients at 80.0 ± 7.3 years and mean age of the informants at 56.7 ± 11.1 years. Mean CDR-SB were 6.4 ± 4.3, which were significantly lower than the first registration. A total of 66.6% of the informants lived with the patients, which was lower than 69.3% in the previous registration. Most patients were right handed (95.3%) and spoke Taiwanese (83%). More patients were widowed during the registration period (45.1% vs 38.2%) (Table 2).

**Table 1.** Demographic Characteristics of Subjects and Informants in Taiwan.

	Subjects			Informant		
	2010-2012	2022-2023	*p*	2010-2012	2022-2023	*p*
Sex, Female, (n/N, %)	446/691, 64.5%	796/1218, 65.4%	0.7	433/690, 62.8%	582/986, 59%	.12
Age, year, n, mean ± SD	n = 690, 79.3 ± 7.7	n = 1217, 80.0 ± 7.3	.093	n = 662, 57.5 ± 13.7	n = 982, 56.7 ± 11.1	0.8
Education, year, n, mean ± SD	n = 677, 6.3 ± 5.1	n = 1218, 7.4 ± 9.6	.074	n = 674, 11.8 ± 4.5	n = 986, 13.7 ± 5.1	<.001
CDR-SB, n, mean ± SD	n = 691, 6.9 ± 3.7	n = 1197, 6.4 ± 4.3	<.001			
MMSE: n, mean ± SD	n = 673, 15.7 ± 6.5	n = 1203, 15.2 ± 7.2	0.2			
			
Living together n/N, %	2010-2012			2022-2023		*p*
	479/691, 69.3%			572/986, 66.6%		<.001

n, number of reported subjects; N, number of recruited subjects; CDR-SB, sum of boxes of the Clinical Dementia Rating scale, MMSE = Mini-Mental Status Examination.

**Table 2.** Handedness, Language and Current Marital Status of the Subjects.

Handedness				First Language				Current Marital Status			
	2010-2012	2022-2023	*p*		2010-2012	2022-2023	*p*		2010-2012	2022-2023	*p*
Left- handed	24/691	33/1218	0.5	Mandarin n/N, %	222/689	176/1218 14.4%	<.001	Married, n/N, %	405/691, 58.6%	629/1218, 51.6%	.031
n/N, %	3.5%	2.7%			32.2%						
Right-handed	654/691	1161/1218		Taiwanese n/N, %	440/689	1016/1218 83%		Widowed, n/N, %	264/691, 38.2%	549/1218, 45.1%	
n/N, %	94.6%	95.3%			63.9%						
Ambidextrous	11/691	23/1218		Hakka n/N, %	21/689	17/1218		Divorced, n/N, %	7/691, 1%	20/1218, 1.6%	
n/N, %	1.6%	1.9%			3%	1.4%					
Unknown handedness	2/691	1/1218		Aboriginal n/N, %	1/689	6/1218		Separated, n/N, %	7/691, 1%	12/1218, 1.0%	
n/N, %	.3%	<.1%			.1%	.5%					
				Others n/N, %	5/689	3/1218		Never married, n/N, %	6/691, .9%	5/1218, .4%	
					.7%	.2%					
								Others, n/N, %	2/691, .2%	3/1218, .2%	

n, number of reported subjects; N, number of recruited subjects.

Table 3 compares the levels of independence and living conditions between the two study periods. More recruited participants lived independently during the present registration period (50% vs 32.5%). More participants lived alone (6.7% vs 6.1%) or with a spouse or partner (47% vs 42.9%) and fewer were admitted to nursing homes (5.2% vs 2.4%).

**Table 3.** Level of Independence and Living Situation of Subjects.

Level of Independence (n/N, %)				Living Situation (n/N, %)			
	2010-2012	2022-2023	*p*		2010-2012	2022-2023	*p*
Independently	223/687, 32.5%	608/1217, 50%	<.001	Alone	42/690, 6.1%	82/1218, 6.7%	.004
Assistance with complex activities	217/687, 31.6%	346/1217, 28.4%		With spouse or partner	296/690, 42.9%	573/1218, 47.0%	
Assistance with basic activities	159/687, 23.1%	160/1217, 13.1%		With other relatives or friends	275/690, 39.9%	483/1218, 39.7%	
Completely dependent	88/687, 12.8%	103/1217, 8.5%		Nursing home	36/690, 5.2%	29/1218, 2.4%	
				Other (eg foreign caregiver)	41/690, 5.9%	51/1218, 4.2%	

For informants who did not live with patients with AD, the frequencies of making in-person visits once daily, once weekly, and once monthly were 32.1%, 20.3%, and 9.2%, respectively, and 26.3%, 14.5%, and 4.3%, respectively, for telephone contact (Table 4). Compared to the 2010-2012 era, the frequency of telephone contact increased significantly in the present registration period.

**Table 4.** Frequency of Contact Between Informants and Subjects not Living Together.

	In-person Visits			Telephone Contact		
	2010-2012	2022-2023	*p*	2010-2012	2022-2023	*p*
Frequency			0.2			<.001
Daily, n/N (%)	60/210, 28.6%	133/414, 32.1%		53/209, 25.4%	109/414, 26.3%	
>3/week, n/N (%)	35/210, 16.7%	98/414, 23.6%		38/209, 18.2%	90/414, 21.7%	
Weekly, n/N (%)	52/210, 24.8%	84/414, 20.3%		42/209, 20.1%	60/414, 14.5%	
>3/month, n/N (%)	22/210, 10.5%	37/414, 8.9%		17/209, 8.1%	20/414, 4.8%	
Monthly, n/N (%)	25/210, 11.9%	38/414, 9.2%		26/209, 12.4%	18/414, 4.3%	
< Monthly, n/N (%)	16/210, 7.6%	24/414, 5.8%		33/209, 15.8%	117/414, 28.3%	

Table 5 shows the family histories of dementia or cognitive impairment among the recruited participants. All information in this study was reported by the informants or patients. A total of 11.2%, 4.1%, and .5% had a reported history of cognitive dysfunction in their mothers, fathers, and children, respectively. A significantly higher percentage of patients with a history of cognitive dysfunction was reported in the patient’s mother than in the first registration (11.2% vs 6.7%). Among the 136 participants with a maternal history of cognitive dysfunction, one reported a diagnosis of MCI (.7%), one had vascular cognitive dysfunction (.7%), two had a behavioral variant of FTD (1.5%), 14 had AD (10.3%), and 118 had dementia of undetermined etiology (86.8%). Among the 50 patients’ fathers with cognitive dysfunction, two reported a diagnosis of MCI (4%), seven reported AD (14%), and 41 reported dementia of undetermined etiology (82%). Among the participants, 10.4% had one sibling with dementia, 1.9% had two siblings with dementia, and .5% had three or more siblings with dementia, which were comparable between the two eras.

**Table 5.** Family History and Number of Dementia or Cognitive Impairment for Subjects in Taiwan.

Percentage of Dementia or Cognitive Impairment in the family	Yes		No		Unknown		*p*
	2010-2012	2022-2023	2010-2012	2022-2023	2010-2012	2022-2023	
Mother, n/N, %	46/691	136/1217	392/61	732/1217	253/691	349/1217	.011
	6.7%	11.2%	56.7%	60.1%	36.6%	28.7%	
Father, n/N, %	19/691	50/1217	415/691	765/1217	257/691	402/1217	0.2
	2.7%	4.1%	60.6%	62.9%	37.2%	33%	
Children, n/N, %	2/674	6/1097	672/674	1091/1097	0	0	0.7
	.3%	.5%	99.7%	99.5%			

Numbers with dementia or cognitive impairment		0	1	2	>3	*p*
Siblings, n/N, %	2010-2012	475/547	59/547	10/547	3/547	0.2
		86.8%	10.8%	1.8%	.5%	
	2022-2023	1018/1167	121/1167	22/1167	6/1167	
		87.2%	10.4%	1.9%	.5%	
Other relatives, n/N, %	2010-2012	490/505	14/505	1/505	0	
		97%	2.8%	.2%		
	2022-2023	Not record				

n, number of reported subjects; N, number of recruited subjects.

Table 6 shows the family histories of other neuropsychiatric disorders in patients with AD. The history of Parkinsonism in their mothers, fathers, children, and siblings was .5%, 1.2%, .2%, and 2.6%, respectively. A history of other neurological problems (99% were stroke) in their mothers, fathers, children, or siblings was 3.7%, 4.6%, 1%, and 8%, respectively. The reported histories of psychiatric disorders among mothers, fathers, children, and siblings were .5%, .2%, 3.9%, and 2.3%, respectively. The patient had no history of ALS recorded.

**Table 6.** Family History and Number of Other Neuropsychiatric Disorders for Subjects in Taiwan. (Only 2022-2023 Data).

Neuropsychiatric Disorders in the family	Parkinsonism	ALS	Other Neurological Problems	Psychiatric Disorders
Mother, n/N, %	6/1217	0	45/1217	6/1217
	.5%		3.7%	.5%
Father, n/N, %	14/1217	0	56/1217	2/1217
	1.2%		4.6%	.2%
Children, n/N, %	2/1097	0	11/1097	43/1097
	.2%		1.0%	3.9%

Numbers of siblings with neuropsychiatric disorders	1	2	3	>3
Parkinsonism	29/1167	1/1167	0	0
	2.5%	.09%		
LS	0	0	0	0
Other neurological problems	84/1167	7/1167	2/1167	0
	7.2%	.6%	.2%	
Psychiatric disorders	23/1167	1/1167	1/1167	1/1167
	2.0%	.09%	.09%	.09%

n, number of reported subjects; N, number of recruited subjects.

## Discussion

This study reports the current status of AD in Taiwan. Compared to a decade ago, AD subjects had milder severity and more of them lived independently, while more informants did not live with the subjects and used more telephone contact.

In response to the rapid growth in the dementia population, the Ministry of Health and Welfare in Taiwan has implemented the Dementia Care Policy since 2013. To further help families with dementia, Taiwan’s government established the Long-Term Care Act 2.0 since 2017, which includes people with dementia over the age of 50.^15^ As part of this reform, the government implemented innovative dementia care programs and settled “Dementia integrated care centers,” which are hospitals or sites that diagnose, provide patient and caregiver education, introduce social resources, promote health literacy of dementia, and educate healthcare providers. As of September 2021, there are 103 dementia-integrated care centers covering all counties in Taiwan. Proper education of clinicians about the diagnosis of cognitive dysfunction and increased health awareness of dementia in the general population both facilitated the earlier identification of subjects with probable or possible AD. The milder severity in the present registration may reflect that people in Taiwan seek medical advice earlier in response to the government’s policy. The frequency of living independently was higher, indicating more patients were diagnosed at the stage of mild cognitive impairment.

Regarding living situation, 86.7% of the participants were living with a spouse, partner, or other relatives (most of them were children). Less AD patients living in nursing homes or with foreign caregivers when compared with the first registration, which may also reflect that the patients were diagnosed earlier. The present study was comparable to the World Alzheimer Report; most patients with dementia were still living at home, and 94% of people living with dementia in low- and middle-income countries were cared for at home.^1^ In addition, 6.7% of patients with AD lived alone, higher than the 6.1% in 2010. People with dementia who live alone are placed into nursing homes earlier because of the potentially increased risk of malnutrition, illness, and fall-related injuries.^16,17^

Of the informants, 66.6% lived with the participants, significantly less than 69.3% in the previous survey. The frequency of in-person and telephone contact increased. Family organizations have changed over the past 50 years in East Asia, with a sharp decline in fertility, later marriages, and non-co-residence with parents.^18^ A couple's economic resources facilitated the breaking away from patrilocal co-residence in Taiwan.^19^

Taiwanese was the most common first language spoken by the subjects, significantly more than at the first registration, because more of the subjects were recruited from middle to southern Taiwan (88% vs 73%), where the most common spoken language is Taiwanese, not Mandarin.

When compared to the UDS3 demographic data reported by the US ADC in 2018, the mean age of our recruited subjects was higher, and they had lower education levels.^9^ There were no family members with known AD or FTD mutations in the present study, compared with .6 and 1.1% in the USA, respectively. However, the USA report included dementia with other etiologies, and 45.7% of the participants had a global CDR score of 0, which made it difficult to compare between studies. Considering only AD patients reported by the ADC in 2005, the participants’ age and sex were similar to those in the present report.^20^

The percentages of patient’s mothers, fathers, and children with dementia were 11.2%, 4.1%, and .5%, respectively. The Rotterdam Study found that 25.6% of patients with AD had first-degree relatives with a history of dementia.^21^ The higher percentage in the Rotterdam cohort may be because each first-degree relative was evaluated by the study physician to confirm a diagnosis of dementia. Female sex is a risk factor for AD, and most subjects recruited in UDS studies were female.^9,20^ The higher percentage of female patients with AD also contributed to a higher percentage of the patients’ mothers having dementia (11.2%) than their fathers (4.1%). Similar trends have been observed in other Asian countries.^7^ The reported maternal history of cognitive dysfunction was significantly higher than that reported during the first registration in Taiwan. The possible explanations were the higher education level of informants to provide a detailed family history and included the diagnosis of both MCI and dementia in the UDS3. The present study may also indicate that the UDS3 provides a more accurate recording of family history through etiological coding.

To the best of our knowledge, no family history of other neurological or psychiatric disorders for patients with AD was reported in Taiwan. Psychiatric history has been suggested to be an integral part of family history, especially in individuals with FTD.^22^ Approximately 40%–50% of patients diagnosed with FTD have a family history of neurological or psychiatric disorders.^23^ Devi et al reported that the incidence of any psychiatric disorder in family members with late-onset AD was 15%.^24^ Further research is needed to determine the family history of these disorders in individuals with dementia.

The strength of this study is its multicenter registration covering all regions of Taiwan, providing an updated clinical picture of patients with AD and their informants compared with 12 years ago. However, this study has some limitations. First, the recruited AD subjects may not be generalizable to all patients with AD in Taiwan owing to selection bias. Second, all family histories were reported by informants or patients, and a reporting bias may exist. It is difficult to determine whether the family histories of dementia are truly indicative of a genetic risk factor for AD. Third, the present study only included patients with clinical diagnosis of AD, therefore, whether the findings are specific to AD or they are also seen in other types of dementia were uncertain. Approximately 30% of the parental history of dementia is unknown, and more than 80% of the etiological diagnoses are uncertain.

## Conclusion

This study updated the demographic data of patients with AD and their informants in Taiwan. Compared to 12 years ago, AD patients were diagnosed at a milder disease stage, and their informants used telephone contact more frequently instead of living with the patients. Family histories of dementia in patients with AD remain frequent. The percentages of patients’ mothers, fathers, siblings, and children who reported having dementia were 11.2%, 4.1%, 12.8%, and .5%, respectively. UDS3.0 help establish a platform for further studies and supports collaborative research.

## Data Availability

According to the nature of the research, supporting data is not available due to ethical restrictions.

## Appendix

### Abbreviations

AD: Alzheimer’s disease
ADC: Alzheimer’s disease centers
ALS: Amyotrophic lateral sclerosis
CASI: Cognitive ability screening instrument
CDR: Clinical dementia rating
CDR-SB: Sum of boxes of the clinical dementia rating scale
FTD: Frontotemporal dementia
MMSE: Mini-mental state examination
MCI: Mild cognitive impairment
UDS: Uniform data set
